# Co‐mast: Harmonized seed production data for woody plants across US long‐term research sites

**DOI:** 10.1002/ecy.4463

**Published:** 2024-12-12

**Authors:** Katherine M. Nigro, Jessica H. Barton, Diana Macias, V. Bala Chaudhary, Ian S. Pearse, David M. Bell, Angel Chen, Natalie L. Cleavitt, Elizabeth E. Crone, David F. Greene, E. Penelope Holland, Jill F. Johnstone, Walter D. Koenig, Nicholas J. Lyon, Tom E. X. Miller, Mark Schulze, Rebecca S. Snell, Jess K. Zimmerman, Johannes M. H. Knops, Stacy McNulty, Robert R. Parmenter, Mark A. Winterstein, Roman I. Zlotin, Jalene M. LaMontagne, Miranda D. Redmond

**Affiliations:** ^1^ Department of Forest and Rangeland Stewardship Colorado State University Fort Collins Colorado USA; ^2^ Department of Biology University of Missouri – St. Louis St. Louis Missouri USA; ^3^ Department of Environmental Science, Policy, and Management University of California Berkeley Berkeley California USA; ^4^ Environmental Studies Department Dartmouth College Hanover New Hampshire USA; ^5^ U.S. Geological Survey Fort Collins Science Center Fort Collins Colorado USA; ^6^ Pacific Northwest Research Station USDA Forest Service Corvallis Oregon USA; ^7^ Long Term Ecological Research Network Office, National Center for Ecological Analysis and Synthesis University of California Santa Barbara Santa Barbara California USA; ^8^ Department of Natural Resources and the Environment Cornell University Ithaca New York USA; ^9^ Department of Evolution & Ecology University of California Davis Davis California USA; ^10^ Department of Forestry, Fire, and Range Management Cal Poly Humboldt Arcata California USA; ^11^ Department of Biology University of York York UK; ^12^ Institute of Arctic Biology University of Alaska Fairbanks Fairbanks Alaska USA; ^13^ Hastings Reservation University of California Berkeley Carmel Valley California USA; ^14^ Department of BioSciences Rice University Houston Texas USA; ^15^ H.J. Andrews Experimental Forest Blue River Oregon USA; ^16^ Department of Environmental and Plant Biology Ohio University Athens Ohio USA; ^17^ Department of Environmental Sciences University of Puerto Rico San Juan Puerto Rico USA; ^18^ Department of Health and Environmental Sciences Xi'an Jiaotong‐Liverpool University Suzhou Jiangsu China; ^19^ Adirondack Ecological Center State University of New York College of Environmental Science and Forestry Newcomb New York USA; ^20^ Department of Biology University of New Mexico Albuquerque New Mexico USA; ^21^ Department of Geography Indiana University Bloomington Indiana USA; ^22^ Whitney R. Harris World Ecology Center University of Missouri – St. Louis St. Louis Missouri USA; ^23^ Science and Conservation Division Missouri Botanical Garden St. Louis Missouri USA; ^24^ Present address: Oak Ridge Institute for Science and Education USA Forest Service, Rocky Mountain Research Station Fort Collins Colorado USA

**Keywords:** community dynamics, long‐term data, LTER, mast fruiting, mast seeding, masting, plant reproduction, plant traits, synchronous reproduction, USA

## Abstract

Plants display a range of temporal patterns of inter‐annual reproduction, from relatively constant seed production to “mast seeding,” the synchronized and highly variable interannual seed production of plants within a population. Previous efforts have compiled global records of seed production in long‐lived plants to gain insight into seed production, forest and animal population dynamics, and the effects of global change on masting. Existing datasets focus on seed production dynamics at the population scale but are limited in their ability to examine community‐level mast seeding dynamics across different plant species at the continental scale. We harmonized decades of plant reproduction data for 141 woody plant species across nine Long‐Term Ecological Research (LTER) or long‐term ecological monitoring sites from a wide range of habitats across the United States. Plant reproduction data are reported annually between 1957 and 2021 and based on either seed traps or seed and/or cone counts on individual trees. A wide range of woody plant species including trees, shrubs, and lianas are represented within sites allowing for direct community‐level comparisons among species. We share code for filtering of data that enables the comparison of plot and individual tree data across sites. For each species, we compiled relevant life history attributes (e.g., seed mass, dispersal syndrome, seed longevity, sexual system) that may serve as important predictors of mast seeding in future analyses. To aid in phylogenetically informed analyses, we also share a phylogeny and phylogenetic distance matrix for all species in the dataset. These data can be used to investigate continent‐scale ecological properties of seed production, including individual and population variability, synchrony within and across species, and how these properties of seed production vary in relation to plant species traits and environmental conditions. In addition, these data can be used to assess how annual variability in seed production is associated with climate conditions and how that varies across populations, species, and regions. The dataset is released under a CC0 1.0 Universal public domain license.

## Supporting information


Data S1:

## Data Availability

The dataset is available as Supporting Information and in Dryad at https://doi.org/10.5061/dryad.69p8cz98q. Code is available in Zenodo at https://doi.org/10.5281/zenodo.10582903.

