# Supplementary material for: Co‐mast: Harmonized seed production data for woody plants across US long‐term research sites
Source: Ecology. 2024 Dec 12;106(1):e4463. doi: 10.1002/ecy.4463 (PMC11739820; doi:10.1002/ecy.4463)
Supplement: Supplementary file 1 — Data S1: [file ECY-106-e4463-s001.zip › MetadataS1.pdf]

Data Paper published in *Ecology*, an Ecological Society of America journal

METADATA S1

Co-Mast: Harmonized seed production data for woody plants across US long term research sites

Katherine M. Nigro[1]; Jessica H. Barton[2]; Diana Macias[3]; V. Bala Chaudhary[4]; Ian S. Pearse[5]; David M. Bell[6]; Angel Chen[7]; Natalie L. Cleavitt[8]; Elizabeth E. Crone[9]; David F. Greene[10]; E. Penelope Holland[11]; Jill F. Johnstone[12]; Walter D. Koenig[13]; Nicholas J. Lyon[7]; Tom E.X. Miller[14]; Mark Schulze[15]; Rebecca S. Snell[16]; Jess K. Zimmerman[17]; Johannes M. H. Knops[18]; Stacy McNulty[19]; Robert R. Parmenter[20]; Mark A. Winterstein[12]; Roman I. Zlotin[21]; Jalene M. LaMontagne[2,23,24]; Miranda D. Redmond[3]

1. Dept. of Forest and Rangeland Stewardship, Colorado State University, Fort Collins, Colorado, USA
2. Department of Biology, University of Missouri – St. Louis, 1 University Blvd, St. Louis, MO, USA, 63121
3. Department of Environmental Science, Policy, and Management. University of California Berkeley, Berkeley, CA, USA.
4. Environmental Studies Department, Dartmouth College, Hanover, NH, USA
5. U.S. Geological Survey, Fort Collins Science Center, Fort Collins, CO, USA
6. Pacific Northwest Research Station, USDA Forest Service, Corvallis, OR
7. Long Term Ecological Research Network Office. National Center for Ecological Analysis and Synthesis, University of California Santa Barbara, Santa Barbara, CA, USA.
8. Department of Natural Resources and the Environment , Cornell University, Ithaca, NY, USA.
9. Department of Evolution & Ecology, University of California, Davis CA 95618 USA
10. Department of Forestry, Fire, and Range Management. Cal Poly Humboldt, Arcata, CA, USA
11. Department of Biology, University of York, York, UK
12. Institute of Arctic Biology, University of Alaska Fairbanks, Fairbanks, AK, USA
13. Hastings Reservation, University of California Berkeley, Carmel Valley, CA, USA
14. Department of BioSciences, Rice University, Houston, TX USA
15. H.J. Andrews Experimental Forest, Blue River, Oregon, USA
16. Department of Environmental and Plant Biology, Ohio University, Athens, OH, USA
17. Department of Environmental Sciences, University of Puerto Rico, San Juan, PR, USA
18. Department of Health and Environmental Sciences, Xi'an Jiaotong-Liverpool University, Suzhou, Jiangsu Province, China

19. Adirondack Ecological Center, State University of New York College of Environmental Science and Forestry, Newcomb, NY, USA
20. Department of Biology, University of New Mexico, Albuquerque, NM, USA
21. Department of Geography, Indiana University, Bloomington, IN, USA (deceased)
22. Whitney R. Harris World Ecology Center, University of Missouri – St. Louis, 1 University Blvd, St. Louis, MO, USA, 63121
23. Science and Conservation Division, Missouri Botanical Garden, 4344 Shaw Blvd., St. Louis, MO, USA, 63110

Corresponding author: Katherine M. Nigro. Email: katienigro83@gmail.com

Current affiliation for Katherine M. Nigro: Oak Ridge Institute for Science and Education; USA Forest Service, Rocky Mountain Research Station, Fort Collins, CO, USA

Authorship note: Roman I. Zlotin, deceased 26 February 2023.

OPEN RESEARCH STATEMENT

The dataset is available as Supporting Information and in Dryad at https://doi.org/10.5061/dryad.69p8cz98q. Code is available in Zenodo at https://doi.org/10.5281/zenodo.10582903.

Data sets utilized for this synthesis are as follows:

Clark, James S., Shannon LaDeau, and Ines Ibanez. 2004. “Fecundity of trees and the colonization–competition hypothesis.” *Ecological monographs* 74, no. 3 (August): 415-442. https://doi.org/10.1890/02-4093.

Fahey, Timothy, and Natalie Cleavitt. 2021. “Tree seed data at the Hubbard Brook Experimental Forest, 1993 - present ver 2.” *Environmental Data Initiative* (January), accessed December 27, 2023. https://doi.org/10.6073/pasta/3d6b29aa80b150e5a9e28a839c05c211.

Franklin, Jerry F. and Mark D. Schulze. 2023. “Cone production of upper slope conifers in the Cascade Range of Oregon and Washington, 1959 to 2022 ver 16.” *Environmental Data Initiative* (March), accessed March 28, 2023. https://doi.org/10.6073/pasta/834405bb4b14582a8f444011f9158740.

Knops, Johannes. 2018. “Acorn production: Acorn survey ver 8.” *Environmental Data Initiative* (June), accessed January 19, 2024. https://doi.org/10.6073/pasta/f856dc4ef3e1ea586bcfb841be7a4700.

McNulty, Stacy A. and Raymond D. Masters. 2019. “Seed production survey, 1988-2009, Adirondack long-term ecological monitoring program project no. 26 by Adirondack Ecological Center of the State University of New York College of Environmental Science and Forestry, Newcomb, New York, USA ver 1.” *Environmental Data Initiative* (June),

accessed January 19, 2024. https://doi.org/10.6073/pasta/f28fe27b04d069dd1f9b4de45488bd8e.

Rapp, Joshua, Elizabeth Crone, and Kristina Stinson. 2023. "Maple reproduction and sap flow at Harvard Forest since 2011 ver 6." *Environmental Data Initiative* (December), accessed January 19, 2024. https://doi.org/10.6073/pasta/7c2ddd7b75680980d84478011c5fbba9.

Van Cleve, Keith, F. Stuart Chapin, Roger Ruess, Michelle C. Mack, and Bonanza Creek LTER. 2022. "Bonanza Creek LTER: Yearly seedfall summary from 1957 to present in the Bonanza Creek Experimental Forest near Fairbanks, Alaska ver 30." *Environmental Data Initiative* (April), accessed January 19, 2024. https://doi.org/10.6073/pasta/373ca46c1df26dc4145bd21ab7e7bb88.

Zimmerman, J. 2022. "Phenologies of the Tabonuco Forest trees and shrubs ver 559507." *Environmental Data Initiative* (June), accessed January 19, 2024. https://doi.org/10.6073/pasta/0fd0832f8619151ab22c8c212357c1c4.

Zlotin, Roman. 2016. "Tree mast production in pinyon-juniper-oak forests at the Sevilleta National Wildlife Refuge, New Mexico (1997- present) ver 154836." *Environmental Data Initiative* (September), accessed January 19, 2024. https://doi.org/10.6073/pasta/f6cb97e094966c0af30206e767b0b2c2.

INTRODUCTION

Mast seeding (or masting), the synchronous and variable production of seed within a population of long-lived plants, is important for the long-term dynamics of forests (Clark et al., 2021) and the population dynamics of the animals that consume seeds (Zwolak et al., 2022) or that prey on seed-eating species (Jensen et al., 2012). Consequently, the management of wildlife populations, timing of silvicultural treatments, and preparation efforts for outbreaks of zoonotic disease can benefit from an improved understanding of the process of masting (Pearse et al., 2021). Recent efforts to compile population-level timeseries of plant reproduction (Hacket-Pain et al., 2022) have been important for stimulating research on the drivers and synchrony of masting between species.

While occurring at a regional scale, masting is fundamentally a population-level phenomenon driven by the synchronous reproduction of individuals within that population. However, many of the proposed consequences of masting should also occur at the community level. For example, the predator satiation hypothesis suggests that variable seed production drives fluctuations in populations of seed eating animals such that a large fraction of a mast crop remains uneaten or cached in "mast" years when the seed crop is large (Janzen, 1971; Fletcher et al., 2010). A key prediction of the predator satiation hypothesis is that plants that produce seeds consumed by the same predators, should mast synchronously, since otherwise generalist predators can simply switch from one species to the other depending on their respective current magnitude of seed crops (Silvertown, 1980; Shibata et al., 1998). There are few publicly available datasets where seed production has been measured on multiple plant species in a community that could be used to test this prediction.

Here we provide a dataset aggregating plant reproduction data on 141 species from 1957 to 2021 across nine environmentally disparate Long-Term Ecological Research (LTER) or long-term ecological monitoring sites in the United States (Fig. 1). Our aim was to create a dataset that harmonizes different sampling methods used to estimate plant reproduction, enabling cross-species comparisons of reproduction across multiple species at sites (i.e., communities) and incorporating attributes of species such as reproductive cycle length, leaf longevity, and mode of dispersal. This complements the data provided in MASTREE+ (Hacket-Pain et al., 2022) by facilitating research on seed production at the community level in woody species with sufficiently long time series (at least 10 years). This focus on community level masting is incorporated in name of this dataset – community masting, or Co-Mast. The data were filtered as described below to eliminate dubious seed production time series, including data from rare species, species whose identification was questionable, and seeds identified only to genus rather than species. These long-term records provide information on seed production that can be used to assess environmental drivers of mast seeding and community level synchrony. Further, when combined with the species phylogenetic and trait data provided here, these data can collectively be used to assess how these factors drive mast seeding across environmental gradients.

Class I. Data Set Descriptors

A. Data set identity: Co-Mast: Harmonized seed production data for woody plants across US long term research sites

B. Data set identification codes:

individual_seed_production.csv
Plot_summarized_seed_data.csv
plot_locations.csv
species_attributes.csv
phylogeny.tr

C. Data set description

1. Principal Investigators: Nigro, Katherine M.; Barton, Jessica; Macias, Diana; Chaudhary, V. Bala; Pearse, Ian S.; Bell, David M.; Chen, Angel; Cleavitt, Natalie; Crone, Elizabeth E.; Greene, David; Holland, E. Penelope; Johnstone, Jill F.; Koenig, Walt; Lyon, Nicholas J.; Miller, Tom E.X.; Schulze, Mark; Snell, Rebecca; Zimmerman, Jess K.; Knops, Johannes M. H.; McNulty, Stacy; Parmenter, Robert R.; Winterstein, Mark A.; Zlotin, Roman I.; LaMontagne, Jalene M.; Redmond, Miranda D.

2. Abstract: Plants display a range of temporal patterns of inter-annual reproduction, from relatively constant seed production to 'mast seeding', the synchronized and highly variable interannual seed production of plants within a population. Previous efforts have compiled global records of seed production in long-lived plants to gain insight into seed production, forest and animal population dynamics, and the effects of global change on masting. Existing datasets focus on seed production dynamics at the population scale, but are limited in their ability to examine community-level mast seeding dynamics across different plant species at the continental scale. We harmonized decades of plant reproduction data for 141 woody plant species across nine Long-Term Ecological Research (LTER) or long-term ecological monitoring sites from a wide range of habitats across the United States. Plant reproduction data are reported annually between 1957 and 2021 and based on either seed-traps or seed and/or cone counts on individual trees. A wide range of woody plant species including trees, shrubs, and lianas are represented within sites allowing for direct community-level comparisons among species. We share code for filtering of data that enables the comparison of plot and individual tree data across sites. For each species, we compiled relevant life history attributes (e.g., seed mass, dispersal syndrome, seed longevity, sexual system) that may serve as important predictors of mast seeding in future analyses. To aid in phylogenetically-informed analyses, we also share a phylogeny and phylogenetic distance matrix for all species in the dataset. These data can be used to investigate continent-scale ecological properties of seed production, including individual and

population variability, synchrony within and across species, and how these properties of seed production vary in relation to plant species traits and environmental conditions. In addition, these data can be used to assess how annual variability in seed production is associated with climate conditions and how that varies across populations, species, and regions. The dataset is released under a CC0 1.0 Universal public domain license.

3. **Keywords:** Community dynamics; LTER; plant reproduction; masting; mast fruiting; mast seeding; synchronous reproduction; long-term data; plant traits; USA
4. **Description:**

individual_seed_production.csv: This file contains measurements of annual seed production for woody plant species from all long-term research sites listed in Table 1. Measurements were taken at multiple individual sampling units (a seed trap or an observation of an individual tree) within a site. Data includes only observations that could be reliably attributed to a particular plant species. Each row contains an observed measurement of seed production for a particular year for a particular species within a sampling unit. We standardized plant taxonomy using the USDA PLANTS database (USDA NRCS, 2024). Seed production was measured in a variety of ways for data of different origins, and the methods of assessing seed production are described for each originating dataset separately under 'Data Descriptors'. Note that counts for data collected via seed traps represent the reproductive structure count per trap while those collected via counts on individual plants represent the reproductive structure count per plant. Seed trap data were not linked to individual trees. In addition, not all sites collected individual count data with the same sampling effort. Therefore, comparisons of actual counts per individual should not be made across sites with differing methodologies. Methods are explained in the "methods_notes" column of this dataset.

plot_summarized_seed_data.csv: This file contains data on seed production summarized to the plot level, using the individual seed production data described above using the "filter_for_data_paper.R" R script. Individual seed production data were filtered to ensure that subsequent analyses would not be biased due to low sampling efforts. To be included, the following criteria had to be met: (1) for seed trap data, the species was observed in ≥ 5% of seed traps; (2) seed production of the species was observed in a minimum of 10 years; (3) fewer than 80% of the years in each time series had 0 seeds/cones/acorns observed; (4) there were at least 4 years with non-zero data in the time series; and (5) for

seed/cone count data, data were collected on at least 10 individual plants. These criteria resulted in 37 species being excluded from the 141 species in the original dataset (individual_seed_production.csv), resulting in 104 species in the filtered dataset.

plot_locations.csv: This file contains the latitude and longitude for each plot in the dataset. Twenty-one of the plots from Andrews Forest did not have digitally available coordinates, and therefore were estimated based on old hand plotted maps, which is noted in the file.

species_atrributes.csv: This file contains aggregated information about species attributes of the 104 plant species present in our filtered dataset. Each row contains observations of species-level attributes. Traits included were: (i) leaf longevity (deciduous, evergreen), ii) dispersal syndrome (abiotic, endozoochory, synzoochory), iii) fleshy fruit (yes, no), iv) growth form (tree, shrub, liana), v) mycorrhizal association (arbuscular, ectomycorrhizal, ericoid, none), vi) pollinator (animal, wind), vii) seed bank (yes, no), viii) seed development time (from bud differentiation to seed maturity: 1 year, 2 years, 3 years), ix) sexual system (dioecious, hermaphrodite, monoecious, polygamo-dioecious), x) shade tolerance (intolerant, intermediate, tolerant), xi) seed mass (mg; continuous variable), xii) leaf type (broadleaf, needleleaf). These data were obtained predominantly from the US woody seed manual (USDA, 2008), the Silvics of North America (Burns & Honkala, 2008), the TRY database (Kattge et al., 2011; 2020), and the USDA Plant Database (USDA NRCS, 2024). Full citations can be found in "attribute-citations.csv".

The information of both plot_summarized_seed_data.csv and species_attributes.csv can be matched by joining by the 'species_name' column.

attribute-citations.csv: This file contains all citations used in compiling the species attribute table.

phylogeny.tr: This file contains a phylogenetic tree for the 104 plant species present in our filtered dataset. The phylogenetic tree was based on Zanne et al. (2014), and 94 of our species matched exactly their phylogeny. Of the remaining 10 species, eight matched a genus on the Zanne et al. (2014) phylogeny, and are placed as polytomies at the genus level. The remaining two species did not match at the genus level, and they were placed as polytomies at the family level. The R script "phylogenetic_tree.r" produced the phylogeny. An alternate method for constructing a phylogenetic tree based on Smith & Brown (2018) is also included at the end of the script.

Class II. Research origin descriptors

B. Specific subproject description

1. **Site descriptions, sampling design, research methods, and personnel**

a. **Geographic description**

Data are from nine Long-Term Ecological Research (LTER) or long-term ecological monitoring sites from a wide range of habitats spanning the United States from Alaska in the north to Puerto Rico in the south (Figure 1). Sites span 46 degrees of latitude and 82 degrees of longitude.

b. **Temporal coverage**

The dataset contains 64 years of data (all sites combined) spanning 1957-2021. Individual sites have varying timespans (Table 1).

c. **Site descriptions**

Adirondack Ecological Center

Stacy A. McNulty (smcnulty@esf.edu), Raymond D. Masters

Adirondack Ecological Center is a long term ecological monitoring site (https://www.esf.edu/aec/research/altemp.php). Seed traps (18.9 L plastic buckets, surface area of 0.0729 m^2) were collected biannually in the spring and fall at Huntington Wildlife Forest at The State University of New York College of Environmental Science and Forestry. Fifty buckets were placed 30 m apart from one another and 0.5 m off the ground using metal stakes in a 350-year-old unmanaged forest with two forest types, deciduous and mixed conifer/deciduous (25 buckets in each forest type). Material from buckets was sorted, seeds were identified down to species, and yearly totals were recorded for each bucket in each season. For our data compilation, we tallied seeds in both spring and fall counts in the same calendar year for species that disperse their seeds in the spring, and tallied the fall bucket of the current year and spring bucket of the following year for species that disperse their seeds in the fall. The raw data can be found in McNulty & Masters (2019).

Andrews Forest (US LTER)

Mark D. Schulze (mark.schulze@oregonstate.edu), Jerry F. Franklin

Sites across the Cascade Mountains in Oregon including Andrews Forest (AND) and Washington (plus a coast range site) were selected in the 1960s, with a few sites added in later years. Plots are single species samples of 20-30 marked trees. In a few cases, “plots” overlap spatially, as two species in the same forest stand were selected but called separate plots. Plots were selected to cover the range of the Cascade Mountains,

and trees within plots were selected to be dominant or codominant individuals with good viewing angles. Trails, roads, natural vegetation breaks and clearcut edges were used to provide good viewing angles. Data are reported as the number of cones for each individual. Observation start and end years vary among plots for several reasons: plots were added to the study in several pulses, with most beginning 1962-1965, but with a subset in the 1970s and 1980s. Plots were dropped due to major wildfires, volcano eruptions and, beginning in 2018, funding shortages. Occasional missed years for a given plot result from access issues due to active major wildfires in the area. Some plots have experienced significant mortality, and new trees have only been added sporadically as funding allows, meaning the number of trees observed is not constant over time. The raw data and metadata can be found in Franklin & Schulze (2023).

Bonanza Creek (US LTER)
Jill Johnstone (jfjohnstone@alaska.edu), Keith Van Cleve, F. Stuart Chapin, Roger Ruess, Michelle C. Mack

Seed production data from Bonanza Creek (BNZ) capture annual variation in seed fall within forest stands. Seed traps (0.5m x 0.5m wooden frames with mesh liners) were placed to collect fallen seed under trees at a site, with traps deployed along transects within a 50m x 60m site. Samples from 1987 onward are based on 3 traps deployed along 2 transects (6 total per site). Seeds were collected in the spring, following snowmelt, and the larger seeds of woody trees and shrubs were counted. Data are reported as the count of seeds in individual traps for the seed production year prior to the collection year, since the monitored species disperse their seeds in fall and winter. Seeds of tree species *Populus balsamifera* and *P. tremuloides*, which may co-occur at sites, are not included in the counts as their seeds are very small and dispersed in spring rather than fall. Sites at BNZ were established to represent different successional stages along a hypothesized sequence of floodplain (primary) and post-fire (secondary) succession. The majority of sites have undergone successional changes in canopy structure over the course of several decades. The period of seed collection began in 1985 or later for most (8) sites. Seed collection for *Picea glauca* began in 1957 at site UP1A and 1969 at UP3A, with varying numbers of seed traps used over time up to 1987. Site UP1A burned in 1983 but seed collection was re-established at the site a few years later. Sites with historic seed collection of *Picea glauca* during the 1970's (FPSH and TS04) were not maintained after program reorganization in the mid-

1980's. The raw data and metadata can be found in Van Cleve et al. (2022).

Cedar Creek Ecosystem Science Reserve (US LTER)
Walt Koenig (wdkoenig@berkeley.edu) and Johannes M. H. Knops (Johannes.Knops@xjtlu.edu.cn)

Data are 30 second visual counts of the number of acorns counted on individual trees at Cedar Creek (CDR), following the protocol of Koenig et al. (1994). Plots were burned in the spring on a schedule that varied among plots, which has been shown to affect acorn production (Funk et al., 2016). The data include whether the plot was burned the prior spring (burned=1); 1 year previously (burned.m1yr=1); or 2 years previously (burned.m2yr=1). The raw data and metadata can be found in Knops (2018).

Coweeta
Jim Clark (jclark@duke.edu), Inés Ibáñez

Seed traps were located at seven forest stands at Coweeta (CWT) at regular intervals. Traps were emptied between 1 and 6 times per year at each plot. For each species, seeds per year were summed by adding the number of seeds found in traps after the month of typical seed maturation in the current year, with all seeds found in traps the following year prior to the month of typical seed maturation (e.g. for a species whose seeds mature in June, the seed count in 2001 would include seeds collected from June 2001 - May 2002). Some seeds were only identified to the genus level, and because of that species-level data is not available for all species, including some of the dominant species. The raw data were provided by Inés Ibáñez and Jim Clark (Clark et al., 2004).

Harvard Forest (US LTER)
Elizabeth Crone (ecrone@ucdavis.edu), Joshua Rapp, Kristina Stinson

Reproduction is evaluated at both the fruiting and flowering stages for *Acer saccharum* at Harvard Forest (HFR). During flowering (April-early May) flowering effort was qualitatively evaluated by number of flowering buds (low: < 1,000, medium: 1,000-10,000, high: > 10,000). Trees were also recorded as having only male flowers or having both female and male flowers. Whole tree seed production was evaluated by visual timed counts

of seeds across the canopy, with two observers counting the number of seeds observed in 15 seconds, and the summed count of these used as a metric for total reproduction for that individual. The raw data and metadata can be found in Rapp et al. (2023).

Hubbard Brook (US LTER)
Nat Cleavitt (nlc4@cornell.edu), Tim Fahey

Adjacent to and within the south-facing watershed area of Hubbard Brook (HBR), seeds are collected via basket style seed traps (0.1 m^2 area) elevated on fence posts 1m above the ground. Seeds, leaves and other fine litter are collected, sorted, and counted three times per "year" (August, November and May of the following calendar year). There are a total of 10 plots with 10-12 baskets at each plot. Plots are distributed across three geographic areas, and stratified by elevation zones (low, mid, upper and high). Two of the geographic areas are reference (or control areas) started in 1993 and the third is a Calcium silicate addition treatment started in 1996 (Cleavitt & Fahey, 2017). The raw data and metadata can be found in Fahey & Cleavitt (2021).

Luquillo (US LTER)
Jess Zimmerman (jesskz@ites.upr.edu)

At the El Verde Field Station, 120 numbered baskets were placed along trails in the 16 ha Luquillo Forest Dynamics Plot (LUQ). Fern and angiosperm flowers and fruits are monitored biweekly. The data from LFDP began in April of 1992. Originally, traps measured 0.16 m^2 but were replaced in 2006 with traps 0.5 m^2. Each reproductive part collected is counted and identified to species using a six-letter code. Reproductive parts are identified with a number code. Counts are summed for each calendar year. The raw data and metadata can be found in Zimmerman (2022).

Sevilleta (US LTER)
Roman I. Zlotin (deceased), Diana S. Macias (dianamacias@berkeley.edu), Robert R. Parmenter (parmentr@unm.edu),

Annual mast fruit production is measured in August at five sites within the Sevilleta National Wildlife Refuge (SEV), beginning in 1997. Three different methods were developed to estimate annual production. For

piñon pine (*Pinus edulis*) estimates are made by visually counting the third-year, ripened, mature cones per tree (n = 210 marked trees) with binoculars and multiplying the # of cones by the mean number of intact seeds per cone to estimate seeds per tree; for Sonoran scrub oak *(Quercus turbinellla*) estimates utilize the number of acorns per 0.1 m^2 of canopy surface area in 3-5 replicates, and scaling up to the size of the entire individual (n = 194 marked trees); and for one-seed juniper (*Juniperus monosperma*) the percent of twigs with berries and the quantity of berries per twig are determined every year for all trees in each plot (n = 412 trees) (Parmenter et al., 2018). The raw data and metadata can be found in Zlotin (2016).

Table 1. Long-term Ecological Research (LTER) and long-term ecological monitoring sites included in the data. Sites listed by latitude sorted from north to south. Several of the sites have multiple plots, some of which can be 100s of km apart and the latitude and longitude for each individual plot is included in "plot_locations.csv". Climate data were extracted from TerraClimate (Abatzoglou *et al.* 2018; 4 km resolution) and represent the mean and range for mean annual temperature and mean annual precipitation (from 1958 - 2021) across all plots.

Site Name	*State or Territory*	*Habitat*	*Latitude & longitude*	*Mean (range) annual temperature (°C)*	*Mean (range) annual ppt (mm)*	*Years in data*	*Seed observation type*	*N plots*	*N species*
Bonanza Creek (BNZ)	AK	Taiga	64.858°N 147.847°W	–2.7 (–3.0 to –2.2)	312.3 (303.3–333.4)	1957–2019	Trap	12	6
Cedar Creek (CDR)	MN	Savanna/ tallgrass prairie	45.401°N 93.201°W	6.6	749.0	1995–2011	Count	7	4
Andrews Forest (AND)	OR and WA	Coniferous forest	44.212°N 122.256°W	6.1 (3.5–11.7)	1774.4 (1069.1–2716.1)	1959–2019	Count	61	10
Adirondack Ecological Center (AEC)	NY	Deciduous/ mixed forest	44.024°N 74.281°W	4.8	1086.6	1988–2009	Trap	50	7
Hubbard Brook (HBR)	NH	Deciduous forest	43.940°N 71.751°W	5.4 (5.3–5.4)	1167.2 (1162.8–1167.6)	1993–2020	Trap	10	3
Harvard Forest (HFR)	MA	Deciduous forest	42.530°N 72.190°W	7.2	1101.8	2011–2021	Count	1	1
Coweeta (CWT)	NC	Deciduous forest	35.000°N 83.500°W	11.5 (10.4–12.3)	1907.3 (1818.6–2025.6)	1991–2019	Trap	7	20
Sevilleta (SEV)	NM	Pinyon - Juniper - Oak woodland	34.353°N 106.882°W	12.7 (11.7–14.0)	266.4 (211.3–323.5)	1997–2019	Count	5	3
Luquillo (LUQ)	PR	Tropical rainforest	18.300°N 65.800°W	21.8	2174.3	1992–2021	Trap	1	95

Class III. Data set status and accessibility
A. Status
Latest update: January 2024
Latest archive date: January 2024
Metadata status: Updated in January 2024, metadata are current.
Data verification: The final data were verified by KN, MR, and BC.
B. Accessibility
1. Storage location and medium: the files 'individual_seed_production.csv', 'plot_summarized_seed_data.csv', 'plot_locations.csv', and 'species_attributes.csv' can be downloaded with associated metadata information (in the Supporting Information) through the ECOLOGY journal and on Dryad (https://doi.org/10.5061/dryad.69p8cz98q). The code file 'filter_for_data_paper.R' can be downloaded from Zenodo (https://doi.org/10.5281/zenodo.10582903). Most original datasets for the LTER and long-term monitoring sites can be found on the Environmental Data Initiative Data Portal. A backup with all the files and codes are stored in the Originators' personal computers and with the National Center for Ecological Analysis and Synthesis. Data adhere to the Findable, Accessible, Interoperable, and Reusable (FAIR) data standards.
2. Contact person: For general inquiries about the database: Katie Nigro (katienigro83@gmail.com). For specific questions on a particular dataset, find the best person to contact for that dataset in Class A 2.1.2.

Class IV. Data structural descriptors

A. Data set file

1. **Identity:** individual_seed_production.csv; plot_summarized_seed_data.csv; plot_locations.csv; species_attributes.csv; phylogeny.tr
2. **File format:** The data files are served in comma-separated value (.CSV) files with UTF-8 encoding.

B. Variable information

Table 2: Definitions and units of columns in the individual seed production data ("individual_seed_production.csv"), which is the unfiltered data.

Column name	Definition	Units
site_name	The name of the LTER/long-term ecological monitoring site	
megaplot	This variable applies to data only from the site Andrews Forest (AND) and Cedar Creek (CDR) and is used to groups plots into "megaplots". Megaplots are designated based on their proximity to each other in order to allow comparisons of cross-species synchrony.	
plot	The name of the unique plot at each site that the data came from. Plots are designated by expert staff as individual forest stands that share environmental characteristics in a given location. This is the same as the megaplot name at all sites except AND and CDR.	
trap	A unique identifier for the trap from which seeds were counted (only applicable to sites where seed traps were used).	
plant_ID	A unique identifier for the plant on which seeds/cones were counted (only applicable to sites where seeds or cones on individual plants were counted).	
species_name	The scientific name of the species. Names are written as Genus.species.	
year	The year in which seeds/cones matured. See individual site descriptions for how this was determined at each site.	

count	Count of reproductive structures (cones, seeds, acorns, etc.) in corresponding seed trap or tree/shrub canopy.	
stem_diameter_cm	Diameter at breast height (DBH) of corresponding plant in corresponding year. Only applicable to AND.	cm
trap_area_m2	Area of seed trap where seeds were collected (only applicable to sites where seed traps were used).	m^2
height_diameter_taken	Typically this will be at breast height (1.4 m), which is listed as "Breast Height" but if done at the root collar for species like pinyon or juniper then it's listed as "Root Collar". Only applicable for sites that measured individual plant data.	
burned	Only applicable to Cedar Creek (CDR) and denotes whether the plot had burned recently in prescribed burn treatments. We note whether the plot burned in the prior spring (burned=1), two years previously (burned =2) or has not burned within the last two years (burned=0).	
general_method	Whether the data was collected with seed traps ("TRAP") or with cone counts ("CONECOUNT") or seed counts ("SEEDCOUNT") on trees. For cone and seed counts that were only done in part of a tree canopy, then "PARTIALCONECOUNT" or "PARTIALSEEDCOUNT" is instead noted. If partial counts were done in tree canopies that were scaled to whole tree by a multiplier then we note that	

	the cone or seed count is estimated, by stating "ESTIMATEDCONECOUNT" or, in the case of estimated seed counts, "ESTIMATEDSEEDCOUNT". If the method was done by counting seeds or cones in a set amount of time, then "TIMEDCONECOUNT" or "TIMEDSEEDCOUNT" is noted.	
methods_notes	Any relevant notes on the method, such as viewing area for counting cones/seeds, type of seed trap used, time in which cones/seeds were counted if timed counts were used, etc.	

Table 3. Metadata for "plot_summarized_seed_data.csv", which is plant reproduction data that met our filtering criteria, summarized at the plot scale. Sites either report data as cone counts or seed trap data, as indicated in Table 1.

Column Name	Description	Range/Levels
site_name	The name of the LTER/long-term ecological monitoring site	9 sites, see Table 1 for site characteristics
plot	Within each site, plots are designated by expert staff as individual forest stands that share environmental characteristics in a given location.	96 plots, ranging from 1–53 at each site.
megaplot	This variable applies to data from the sites Andrews Forest (AND) and Cedar Creek (CDR) and is used to groups plots into "megaplots". Megaplots are designated based on their proximity to each other in order to allow comparisons of cross-species synchrony.	61 megaplots, ranging from 1–24 at each site.
species_name	The scientific name of species. Names are written as Genus.Species. Matches the "species_name" column in all other data files.	104 woody plant species, full list of species is given in species_attributes.csv
year	Year in which seeds/cones matured. See individual site descriptions for how this was determined at each site.	1957–2021
total_trap_area_m2	Total trap area (summed) in a given year at the plot scale, expressed in units of m^2. Each trap has an area that it is collecting data from.	0.1133–60.00 m^2/plot
seeds_per_m2	Total number of seeds divided by total trap area in m^2 per plot. In a given year.	0.00–67,320.49 seeds/m^2
total_seeds	Total number of seeds per plot in a given year	0.00–1,120,213.00 seeds/plot
total_traps	Total number of traps per plot.	1–120 traps/plot

	Only applies to sites that use trap data (see Table 1).	
seeds_or_cones_per_tree	Total number of seeds or cones per plot divided by the total number of trees. Only applies to sites that use individual seed/cone count data (see Table 1).	0.00–1,588.89 seeds or cones/tree
total_seeds_or_cones	Total number of seeds or cones per plot in a given year. Only applies to sites that use individual seed/cone count data (see Table 1).	0.00–28,600.00 seeds or cones/plot
total_trees	Total number of trees per plot that were included in seed or cone counts. Only applies to sites that use individual seed/cone count data (see Table 1).	0–219 trees/plot
collections_per_yr	The number of times per year that seed traps or individual seed production were counted. This varies by site.	1–26 collections/year
plot_lat	Latitude of plot in units of decimal degrees	18.33° to 65.15°
plot_long	Longitude of plot in units of decimal degrees	−148.36° to −65.82°

Table 4. Metadata for "species_attributes.csv", which lists all species included in the plot-level data along with some of their primary attributes or traits. Data predominantly came from the US woody seed manual (USDA, 2008), the Silvics of North America (Burns and Honkala, 2008), the TRY database (Kattge et al., 2011; 2020), and the USDA Plant Database (USDA NRCS, 2022). Additional data sources that were used and each data source that populated the TRY database are provided in the CSV file: "attribute-citations.csv". For all attributes we used expert knowledge from members of this team to ensure that data reported is consistent with observed species attributes in the field.

**Name**	**Description**	**Range / Levels**
species_name	The scientific name of each species, matches the "species_name" column in all other data files	
family	The family of each species	
genus	The genus of each species	
epithet	The epithet of each species	
seed_development_years	Time (in years) for seed development, from seed initiation to mature seed development in years (1 = 12 months or less; 2 = 13–24 months; 3 = 25–36 months).	1–3 years
pollinator_code	Primary pollination vector (animal or wind). Animal pollinators included insects and birds.	animal,<br>wind
mycorrhiza_type	The dominant type of plant-fungal mycorrhizal symbiosis for each species.	EM, AM, Ericoid, none
needleleaf_broadleaf	Whether the leaf form is needleleaf or broadleaf	Needleleaf,<br>Broadleaf
deciduous_evergreen	Whether the species is deciduous or evergreen	Deciduous,<br>Evergreen
seed_maturation_timing	Time period of seed maturation. These were broken into dominant season(s),	Fall, Winter,<br>Spring, Summer,

	based on the month(s) of reported seed maturation. Seasons were defined as: Summer (June, July, August), Fall (September, October, November), winter (December, January, February), and Spring (March, April, May), with late summer being included as an additional category since many species reproduced during the August-September months. Some species reproduce in multiple seasons (e.g. Summer - Fall) and others can reproduce at multiple distinct times of the year (e.g. Spring and Fall). If a species reproduced across seasons, then only the season where most reproduction occurred is noted.	Late Summer, Late Summer–Fall, Summer–Fall, Summer–Winter, Winter–Spring, Spring–Summer, Spring & Fall, Fall–Summer, Fall–Winter, Fall–Spring, Winter–Summer, Summer & Fall, Winter & Fall, All Year
seed_mass_mg	Average seed mass (in mg per seed).	0.019–6044 mg
sexual_system	The production of pollen and ovules, when produced on separate individuals (Dioecious), or the same individual but in separate structures (Monoecious), or within the same structure (Hermaphrodite). Polygamodioecious refers to species that can have both single-sex and bisexual flowers on the same individual.	Monoecious, Dioecious, Hermaphrodite, polygamo-Dioecious
shade_tolerance	A species shade tolerance level, where Tolerant = shade tolerant; Intermediate = intermediate shade tolerance; and Intolerant = shade intolerant	Tolerant, Intermediate, Intolerant
growth_form	The species dominant growth form	Tree, Shrub, Liana
seed_bank	If seeds can remain viable for over a year, either in the canopy or in the soil, then the species is considered to seed bank and given a “yes”.	yes, no

fleshy_fruit	Ovary wall succulent, not hard and dry	yes, no
dispersal_syndrome	The mechanisms by which seed dispersal occurs. Abiotic includes wind, water, and gravity dispersal mechanisms whereas animal dispersal mechanisms are those broken down by endozoochory and synzoochory.	abiotic, endozoochory, synzoochory

Table 5. Metadata for "plot_locations.csv", which provides the latitude and longitude of each plot in the 'individual_seed_production.csv' file.

Column Name	Description	Range/Levels
site_name	The name of the LTER/long-term ecological monitoring site	9 sites, see Table 1 for site characteristics
plot	Within each site, plots are designated by expert staff as individual forest stands that share environmental characteristics in a given location.	105 plots, ranging from 1–61 at each site.
megaplot	This variable applies to data from the sites Andrews Forest (AND) and Cedar Creek (CDR) and is used to groups plots into "megaplots". Megaplots are designated based on their proximity to each other in order to allow comparisons of cross-species synchrony.	63 megaplots
Latitude_dd	Latitude of plot in units of decimal degrees	18.33° to 65.15°
Longitude_dd	Longitude of plot in units of decimal degrees	−148.36° to −65.82°
notes	Notes about how coordinates were acquired, if not originally present in the data	NA or "coordinates estimated from hand plotted map"

Acknowledgements

This data harmonization is a product of the Identifying environmental drivers of plant reproduction across LTER sites' synthesis working group, Long-Term Ecological Research Network Office (LNO), National Center for Ecological Analysis and Synthesis (NCEAS) through NSF award # 1929393. This group was led by JML, EEC, and MDR, and all authors across a range of institutions and career stages contributed to this work during remote and in-person working group meetings that began during the COVID-19 pandemic. We acknowledge support from the LNO at NCEAS including Marty Downs, Ginger Gilquist, and Michelle Morris, and we thank all of the researchers who collected the long-term field data at LTER sites that were used in this data product. We also thank Jim Clark and Inés Ibáñez for providing seed production data from Coweeta. This work was supported by National Science Foundation grants (DEB-1122325, DEB-1754435, DEB-1546686, DEB-2025755, DEB-1114804, DEB-1637685).

This material is based upon work supported by the H.J. Andrews Experimental Forest and Long Term Ecological Research (LTER) program under the NSF grant LTER8 DEB-2025755. Hubbard Brook is part of the LTER network, which is supported by the U.S. National Science Foundation. Hubbard Brook Experimental Forest is operated and maintained by the U.S. Department of Agriculture, Forest Service, Northern Research Station. Bonanza Creek is supported by the National Science Foundation (DEB-2224776 and DEB-1636476) and by the USDA Forest Service, Pacific Northwest Research Station (RJVA-PNW-20-JV-11261932-018). Any use of trade, firm, or product names is for descriptive purposes only and does not imply endorsement by the U.S. Government.

Conflict of Interest Statement:
The authors declare no conflicts of interest.

References

Abatzoglou, John T., Solomon Z. Dobrowski, Sean A. Parks, and Katherine C. Hegewisch. 2018. "TerraClimate, a high-resolution global dataset of monthly climate and climatic water balance from 1958–2015." *Scientific data* 5, no. 170191 (January): 1-12. https://doi.org/10.1038/sdata.2017.191.

Burns, Russell M., and Barbara Honkala. 1990. *Silvics of North America*. Washington, D.C., USA: U.S. Department of Agriculture.

Clark, James S., Robert Andrus, Melanie Aubry-Kientz, Yves Bergeron, Michal Bogdziewicz, Don C. Bragg, Dale Brockway, et al. 2021. "Continent-wide tree fecundity driven by indirect climate effects." *Nature communications* 12, no. 1242 (February): 1-11. https://doi.org/10.1038/s41467-020-20836-3.

Clark, James S., Shannon LaDeau, and Ines Ibanez. 2004. "Fecundity of trees and the colonization–competition hypothesis." *Ecological monographs* 74, no. 3 (August): 415-442. https://doi.org/10.1890/02-4093.

Cleavitt, Natalie L., and Timothy J. Fahey. 2017. "Seed production of sugar maple and American beech in northern hardwood forests, New Hampshire, USA." *Canadian Journal of Forest Research* 47, no. 7 (April): 985-990. https://doi.org/10.1139/cjfr-2017-0096.

Fahey, Timothy, and Natalie Cleavitt. 2021. "Tree seed data at the Hubbard Brook Experimental Forest, 1993 - present ver 2." *Environmental Data Initiative* (January), accessed December 27, 2023. https://doi.org/10.6073/pasta/3d6b29aa80b150e5a9e28a839c05c211.

Fletcher, Quinn E., Stan Boutin, Jeffrey E. Lane, Jalene M. LaMontagne, Andrew G. McAdam, Charles J. Krebs, and Murray M. Humphries. 2010. "The functional response of a hoarding seed predator to mast seeding." *Ecology* 91, no. 9 (September): 2673-2683. https://doi.org/10.1890/09-1816.1.

Franklin, Jerry F. and Mark D. Schulze. 2023. "Cone production of upper slope conifers in the Cascade Range of Oregon and Washington, 1959 to 2022 ver 16." *Environmental Data*

Initiative (March), accessed March 28, 2023. https://doi.org/10.6073/pasta/834405bb4b14582a8f444011f9158740.

Funk, Kyle A., Walter D. Koenig, and Johannes M. H. Knops. 2016. “Fire effects on acorn production are consistent with the stored resource hypothesis for masting behavior.” *Canadian Journal of Forest Research* 46, no. 1 (October): 20–24. https://doi.org/10.1139/cjfr-2015-0227.

Hacket-Pain, Andrew, Jessie J. Foest, Ian S. Pearse, Jalene M. LaMontagne, Walter D. Koenig, Giorgio Vacchiano, Michal Bogdziewicz, et al. 2022. “MASTREE+: Time-series of plant reproductive effort from six continents.” *Global change biology* 28, no. 9 (February): 3066-3082. https://doi.org/10.1111/gcb.16130.

Janzen, Daniel H. 1971. “Seed predation by animals.” *Annual Review of Ecology and Systematics* 2: 465–492.

Jensen, Paul G., Charlotte L. Demers, Stacy A. McNulty, Walter J. Jakubas, and Murray M. Humphries. 2012. “Marten and fisher responses to fluctuations in prey populations and mast crops in the northern hardwood forest.” *Journal of Wildlife Management* 76, no. 3 (January): 489–502. https://doi.org/10.1002/jwmg.322.

Kattge, Jens, Gerhard Bönisch, Sandra Díaz, Sandra Lavorel, Iain C. Prentice, Paul Leadley, Susanne Tautenhahn, et al. 2020. “TRY plant trait database - enhanced coverage and open access.” *Global Change Biology* 26, no. 1 (January): 119–188. https://doi.org/10.1111/gcb.14904.

Kattge, Jens, Sandra Díaz, Sandra Lavorel, Iain C. Prentice, Paul Leadley, Gerhard Bönisch, Eric Garnier, et al. 2011. “TRY - A global database of plant traits.” *Global Change Biology* 17, no. 9 (April): 2905–2935. https://doi.org/10.1111/j.1365-2486.2011.02451.x.

Knops, Johannes. 2018. “Acorn production: Acorn survey ver 8.” *Environmental Data Initiative* (June), accessed January 19, 2024. https://doi.org/10.6073/pasta/f856dc4ef3e1ea586bcfb841be7a4700.

Koenig, Walter D., Johannes M. Knops, William J. Carmen, Mark T. Stanback, and Ronald L. Mumme. 1994. “Estimating acorn crops using visual surveys.” *Canadian Journal of Forest Research* 24, no. 10 (October): 2105-2112. https://doi.org/10.1139/x94-270.

McNulty, Stacy A. and Raymond D. Masters. 2019. “Seed production survey, 1988-2009, Adirondack long-term ecological monitoring program project no. 26 by Adirondack Ecological Center of the State University of New York College of Environmental Science and Forestry, Newcomb, New York, USA ver 1.” *Environmental Data Initiative* (June), accessed January 19, 2024. https://doi.org/10.6073/pasta/f28fe27b04d069dd1f9b4de45488bd8e.

Parmenter, Robert R., Roman I. Zlotin, Douglas I. Moore, and Orrin B. Myers. 2018. “Environmental and endogenous drivers of tree mast production and synchrony in piñon-juniper-oak woodlands of New Mexico.” *Ecosphere* 9, no.8 (August): e02360. https://dx.doi.org/10.1002/ecs2.2360.

Pearse, Ian S., Andreas P. Wion, Angela D. Gonzalez, and Mario B. Pesendorfer, 2021.

"Understanding mast seeding for conservation and land management." *Philosophical Transactions of the Royal Society B* 376, (June): 20200383. https://doi.org/10.1098/rstb.2020.0383

Rapp, Joshua, Elizabeth Crone, and Kristina Stinson. 2023. "Maple reproduction and sap flow at Harvard Forest since 2011 ver 6." *Environmental Data Initiative* (December), accessed January 19, 2024. https://doi.org/10.6073/pasta/7c2ddd7b75680980d84478011c5fbba9.

Shibata, Mitsue, Hiroshi Tanaka, and Tohru Nakashizuka. 1998. "Causes and consequences of mast seed production of four co-occurring *Carpinus* species in Japan." *Ecology* 79, no.1 (January): 54-64.

Silvertown, Jonathan W. 1980. "The evolutionary ecology of mast seeding in trees." *Biological Journal of the Linnean Society* 14, no. 2 (September): 235–250. https://doi.org/10.1111/j.1095-8312.1980.tb00107.x.

USDA NRCS, 2024. "The PLANTS Database." National Plant Data Team, Greensboro, NC USA, accessed January 12, 2024. http://plants.usda.gov.USDA, 2008. *The woody plant seed manual*. United State Department of Agriculture, Fort Service, Agriculture Handbook 727.

Van Cleve, Keith, F. Stuart Chapin, Roger Ruess, Michelle C. Mack, and Bonanza Creek LTER. 2022. "Bonanza Creek LTER: Yearly seedfall summary from 1957 to present in the Bonanza Creek Experimental Forest near Fairbanks, Alaska ver 30." *Environmental Data Initiative* (April), accessed January 19, 2024. https://doi.org/10.6073/pasta/373ca46c1df26dc4145bd21ab7e7bb88.

Zanne, Amy E., David C. Tank, William K. Cornwell, Jonathan M. Eastman, Stephen A. Smith, Richard G. FitzJohn, Daniel J. McGlinn, et al. 2014. "Data from: Three keys to the radiation of angiosperms into freezing environments." *Dryad* (October), accessed January 30, 2024. https://doi.org/10.5061/dryad.63q27.

Zimmerman, J. 2022. "Phenologies of the Tabonuco Forest trees and shrubs ver 559507." *Environmental Data Initiative* (June), accessed January 19, 2024. https://doi.org/10.6073/pasta/0fd0832f8619151ab22c8c212357c1c4.

Zlotin, Roman. 2016. "Tree mast production in pinyon-juniper-oak forests at the Sevilleta National Wildlife Refuge, New Mexico (1997- present) ver 154836." *Environmental Data Initiative* (September), accessed January 19, 2024. https://doi.org/10.6073/pasta/f6cb97e094966c0af30206e767b0b2c2.

Zwolak, Rafal, Paulina Celebias, and Michal Bogdziewicz. 2022. "Global patterns in the predator satiation effect of masting: A meta-analysis." *Proceedings of the National Academy of Sciences* 119, no.11 (January): e2105655119. https://doi.org/10.1073/pnas.2105655119.

Class V. Supplemental descriptors

Table 6. Changes made to species identities based on local knowledge from Inés Ibáñez (Coweeta) and Jill Johnstone (Bonanza Creek). The names from the original data are in the "original" column, the names they got changed to are in the "new" column and the reason for doing so is in the "Reason" column.

Site	Original	New	Reason
CWT	*Acer* spp	*Acer rubrum*	Prior to 2010 *Acer* seeds were pooled at the genus level in the seed trap data. Because most of these seeds were *Acer rubrum*, we pooled them with *Acer rubrum* for analysis. For reference, the maximum seeds/trap after 2010 at these sites was 0.1 for *Acer saccarum* and 0.25 for *Acer pensylvanicum* vs. 30 for *Acer rubrum*)
CWT	*Acer pensylvanicum*	Removed	Very low abundance and not identified to species on a regular basis.
CWT	*Acer saccharum*	Removed	High-elevation plots with substantial *Acer saccharum* abundance were added in 2001. *Acer saccharum* seeds were rare in the low-elevation plots that were monitored prior to 2001 and was not consistently identified to species.
CWT	*Amelanchier* spp	*Amelanchier arborea*	This was the only *Amelanchier* species at the site based on stand structure data.
CWT	*Betula* spp, *Betula alleghaniensis* & *Betula lenta*	Removed	These species could not be resolved/separated based on stand structure data.
CWT	Carya spp	*Carya glabra* in plots CWT_318 and CWT_427, otherwise removed	Identified to species in these plots based on stand structure data; these were the plots with only one species.
CWT	*Ilex* spp	*Ilex montana* in plots CWT_427, CWT_527, CWT_LG & CWT_UG, otherwise removed	Identified to species in these plots based on stand structure data; these were the plots with only one species.

CWT	*Magnolia* spp	*Magnolia acuminata* in plots CWT_318 and CWT_527, otherwise removed	Identified to species in these plots based on stand structure data; these were the plots with only one species.
CWT	*Morus* spp	Removed	This species had only a few individual trees. Our guess is that the seed trap data reflect only one tree (I. Ibanez, pers. comm.)
CWT	*Pinus* spp & *Pinus rigida*	Removed	There is a nearby pine plantation where these seeds most likely came from.
CWT	*Prunus* spp	Removed	This species had only a few individual trees. Our guess is that the seed trap data reflect a small number of individuals (I. Ibanez, pers. comm.)
CWT	All *Quercus*	Removed	These species could not be resolved/separated based on stand structure data.
CWT	*Ulmus* spp.	Removed	This genus is rare/absent at Coweeta but could be confused with *Betula*.
CWT	*Vitis* spp.	Removed	Not identified to species in seed traps, and not in stand structure data because it is a vine.
CWT	*Viburnum* spp.	Removed	Not identified to species in seed traps, and not likely to be fully represented in stand structure data (often a small shrub). Possibly not consistently identified in seed trap data (I. Ibanez, pers. comm.)
BNZ	*Alnus* spp.	*Alnus incana* in plots FP1A, FP2A, & FP3A, otherwise *Alnus viridis*	Identified to species in these plots based on stand structure data; these were the plots with only one species.
BNZ	*Betula* spp.	*Betula neoalaskana*	This was the only *Betula* species at the site.

Figures

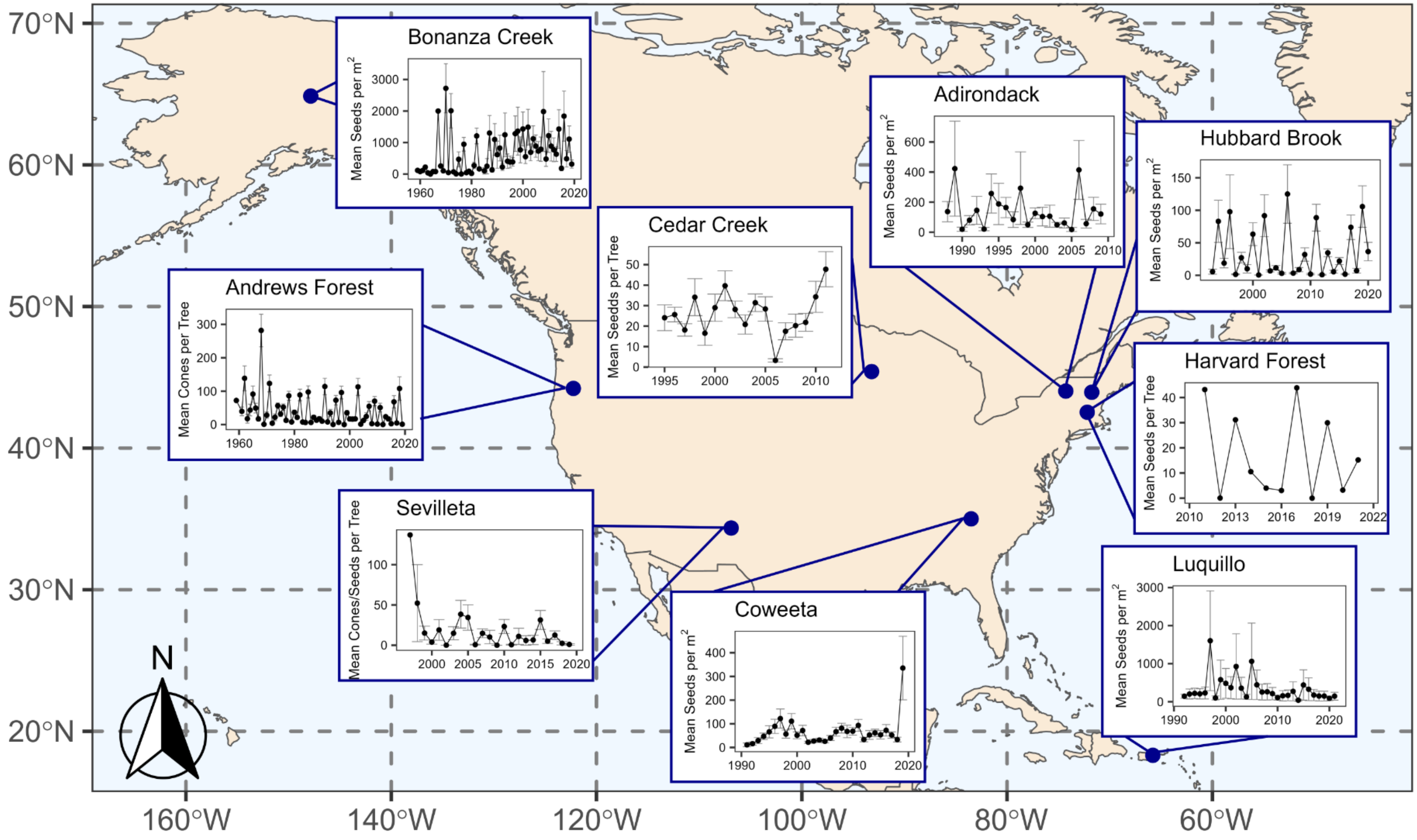

Figure 1. Map of the nine long term ecological research and monitoring sites distributed across North America that are included in the dataset. For sites with seed traps (AEC, BNZ, CWT, HBR, LUQ), figures display average seeds per square meter (points) and standard error (error bars) across plots for all species. For sites with individual counts (AND, CDR, HFR, SEV), figures display average cones or seeds per tree (points) and standard error (error bars) across plots for all species.

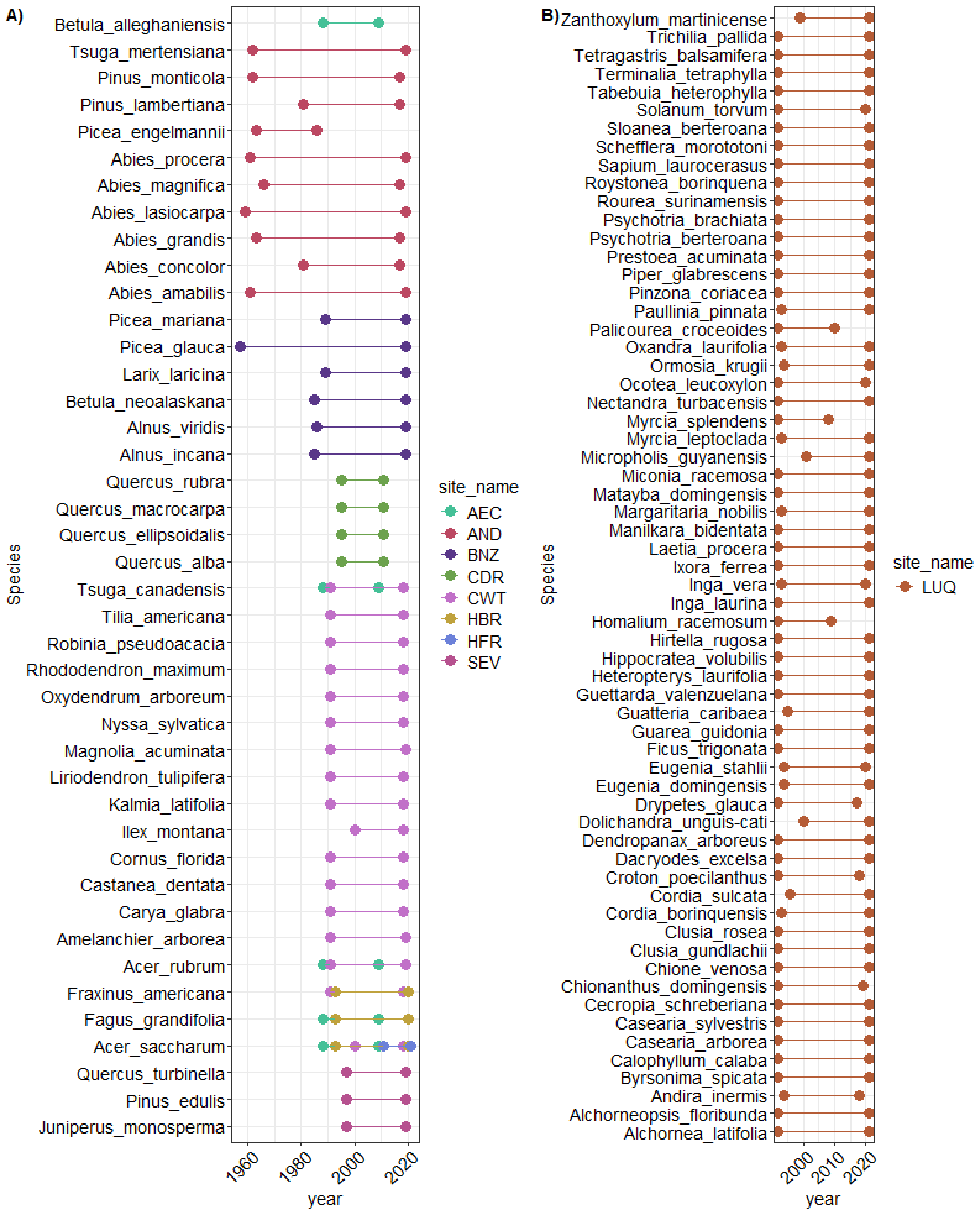

Figure 2. Years of data for each species in the filtered dataset (n = 104; "plot_summarized_seed_data.csv") at each site (n = 9). The Luquillo site (LUQ) is shown separately in panel B due to the large number of species, while all other sites are shown in panel A.
